# Scientific Research Conformity of University Teachers: Role of Incentives and Internal Attribution

**DOI:** 10.3390/brainsci12101302

**Published:** 2022-09-27

**Authors:** Guandong Song, Bin Xiao, Sihui Wang

**Affiliations:** 1 School of Humanities and Law, Northeastern University, Shenyang 110819, China; 2 School of Music, Soochow University, Suzhou 215031, China

**Keywords:** scientific research conformity (SRC), internal attribution type, abidance, compliance, obedience, incentive, model

## Abstract

Background: Conformity is a process by which a person changes his original idea and attitude in response to group pressure and chooses to be consistent with the majority. The study was undertaken to explore university teachers’ scientific research conformity (SRC), the psychological process of information processing and the types of internal attribution in SRC, and the relationship between SRC incentives and types of internal attribution. Method: A survey of 349 teachers from seven universities was conducted. We employed the mixed-method approach; data was collected through in-depth interviews and were analyzed using AMOS. Results: In-depth interviews revealed that the basic principle of human organizational behavior is conformity with incentive, and conformity is a motivational behavior produced through psychological processing of social information. Factor analysis results revealed that teachers’ SRC is characterized by abidance, compliance, and obedience. The internal incentives mainly affect abidance, and have a significant impact on obedience and compliance; the environmental incentives mainly affect abidance, and have a significant impact on obedience. Family and social relationship incentives mainly affect compliance, but social relationship incentives also have a significant impact on obedience. Additionally, policy incentives have a highly significant impact on obedience. Conclusions: This study provides first-hand empirical data for studies pertaining to research motivation and SRC behavior of Chinese university teachers. It also provides a theoretical basis for subsequent research on conformity behavior.

## 1. Introduction

Researchers began researching conformity as early as in 1759. In his “Theory of Moral Sentiments” [1], Adam described conformity as “herding,” that is, a kind of “mechanical imitation,” an irrational and unconscious social contagion [2] or “instinctive imitation” [3,4], also known as “herd behavior” [5]. Most scholars believe that conformity is the process by which a person changes his original idea and attitude in response to group pressure and chooses to be consistent with the majority [6,7]. People change their opinions to conform to the majority [8]. It is also customary to refer to herding behavior because conformity results from group pressure [9]. However, experimental studies show that there is a degree of conformity in case of group size being one or two [10], but when it increases to more than five people, the herding behavior does not increase [11]. Therefore, Moscovici adopted a different approach and proposed the concept of minority influence [12]. Experiments conducted in 1969 suggested that uncertainty caused by social conflict is likely to lead to majority and minority holding the same view [13]. This is contradictory [14], as two opposing causes cannot produce the same phenomenon (i.e., external factors can affect conformity, but cannot explain the nature of conformity). For this reason, we propose internal attribution according to nature [15].

Currently, research on SRC behavior pertains to two aspects: contextual attribution and internal attribution. Intra-attribution is not exactly the same as traditional contextual attribution research [16]. SRC behavior with a contextual attribution perspective refers to the fact that the university teachers exhibit consistent research behaviors under the influence of external pressure [17]. Based on the contextual attribution studies pertaining to conformity research [18], it can be observed that as per the mainstream view, SRC behavior is influenced by external factors [19], as well as by the external context in which the individual is placed, in addition to normative scenarios [20]. These studies provide important implications for advancing the theory and practice of compliance contextual attribution in the context of behaviorist theory, but psychological research must be based on the personality and traits, as individual’s internal factors, as the basis of research, and on the unconscious automatic processes and controllable conscious processes within the individual as the starting point of research [21], in order to reflect the processing of external information within and to indicate the behavior of an individual [22]. In contrast, SRC behavior explained by the internal attribution perspective refers to the fact that university teachers are likely to be influenced by internal and external information to produce different research motivation behaviors.

As early as in 1920s and 1930s, scholars were concerned about the importance of conformity research, conducted numerous experiments and drew inferences to verify the importance of conformity research. According to Allport, “cluster” conveys a kind of trust, not an implication for individuals in irrational situations. Scholars such as Sherif confirmed the occurrence of conformity behavior based on vague and uncertain contexts [23], and scholars such as Asch confirmed the occurrence of conformity behavior in certain contexts [24]. In the case of the experiment that was carried out to determine the conformity behaviors that are likely to arise in ambiguous contexts, Sherif [25] believed the subject could not be sure of their own opinions. Although he had obtained some information, it was very vague. The situation he found at this time seemed to be unquestionable. This is not just an example of the subject’s conformity, as he felt that others had more information. While Asch wanted to demonstrate that social conformity is not always the result of blind and automatic conformity resulting from opinions of the majority. After the test, his conversations with each student revealed that those who developed conformity showed three types of distortion: perception distortion, judgment distortion, and behavior distortion. In recent years, scholars have started validating the findings of Asch and Sherif’s experiment, and hence some scholars are focusing on exploring the value of conformity in college education [26] and college students’ learning [27]. The latest study of the author proposes that college students’ learning conformity behavior refers to internal attribution of social information by individuals [28]. However, few scholars have focused on conformity with respect to the research behavior of university teachers. Therefore, this paper intends to verify the significance of internal attribution of SRC behavior and the value of SRC research in promotion of college teachers’ research abilities.

Recently, scholars have started focusing on defining the subcomponents of conformity studies. Kassin Saul believed that individuals are likely to be affected by different social pressures, and classify herding behavior into conformity, compliance, and obedience [29]. Conformity is an ignorant and automatic process that chooses behavior that is consistent with others in the face of stress. Compliance is a change in behavior that results because of requests from others. According to Kelman, 1958, conformity can be divided into compliance and acceptance. Compliance is a public expression of support for group norms or behaviors, but it does not change individuals’ attitudes and behaviors. Acceptance is the internalization of group norms and behaviors, in the form of guidelines and standards [30]. In 2000, Nail put forward a fusion point of view [31]. According to him conformity can be of three types: compliance, obedience, and acceptance. Compliance does not include the aspect of sincerity but only concerns obeying demands of others. Obedience involves compliance to a definite order to obtain a reward or avoid punishment. Acceptance is heartfelt obedience to others. In addition, the findings of the latest research conducted by the authors have classified college students’ learning conformity as learning compliance, learning abidance, and learning obedience [28]. Based on the conceptual connotation of learning conformity [32], it can be seen that the concept of SRC also focuses on exploring the inner psychological rules pertaining to college teachers’ research behavior, which reflects the differences in their research motivation that has practical significance for enhancing college teachers’ research motivation, thereby improving their research output. Additionally, this paper explores the specific classification of SRC behaviors and the multiple trigger mechanisms.

Despite a long history of research on conformity, the research on SRC is still at an initial stage. Therefore, it is essential to explore the laws of the SRC formation process and the types of internal attribution. Our study analyzes university teachers’ SRC, exploring the psychological process of information processing and the types of internal attribution in SRC, and analyzes the relationship between incentives of SRC and types of internal attribution. The research article comprises five sections: Section 1 introduces the history and origin of internal attribution on conformity. The propositions and hypotheses of SRC have been discussed in Section 2. Section 3 presents detailed information pertaining to methodology, which involved in-depth interviews for analyzing the motivation and the types of internal attribution of SRC. Factor analysis used to verify the types of internal attribution and incentives for constructing the structural equation model on SRC has been illustrated in Section 4. Finally, the results are presented in Section 5.

## 2. Research Propositions and Hypotheses

### 2.1. Research Propositions

The author repeated the Milgram’s field experiment and added interview items to the subjects [33]. It was found that among the 56 participants interviewed after conformity had been established, reasoning provided by the experimental assistant facilitated development of conformity behavior in 47 participants, while in the case of the remaining nine participants, these were just simple instincts or signal conditions [34]. While some participants were influenced by external information, others were influenced by internal information. Furthermore, the authors’ latest research results also point out that university students’ learning conformity behavior is a result of mental processing of social information by individuals, and social information includes both internal and external information [32]. So, a hypothesis pertaining to the interaction of internal and external causes of conformity is proposed: conformity is a motivational behavior produced by people’s psychological processing of social information.

**Proposition** **1.** *SRC is a motivational behavior formed after people process social information.*

According to previous research [31], conformity can be of three types based on motivation, cognitive, emotional, and utilitarian aspects of behavior: abidance, compliance, and obedience.

**Proposition** **2.** *On the basis of motivation, SRC can be divided into three types: abidance, compliance, and obedience.*

According to the level of psychological processing of social information, conformity motivation is divided into explicit motivation and implicit motivation. Motivation is a need or desire that facilitates goal-seeking behavior, and can be divided into two states: instinctive and learned [35]. Instinctive motivation is influenced by genetic or evolutionary causes; it is a kind of self-motivation and can be formed without conscious control. Acquired motivation can be divided into two kinds according to the level of mental processing: (a) habitual motivation, which is influenced by mental stereotypes and is also a kind of self-motivation, (b) conscious motivation, which is formed by complex mental processing of social information (including human memory information) [36]. Self-motivation generated due to instincts or habits can be referred to as implicit motivation, and the motivation formed by complex mental processing can be referred to as explicit motivation [37]. Implicit motivation system originates from an individual’s emotional experience, in combination with the motivation provided by the activity that influences the behavior and facilitates retention of spontaneous behavioral tendencies over time [38]. 

**Figure 1:**
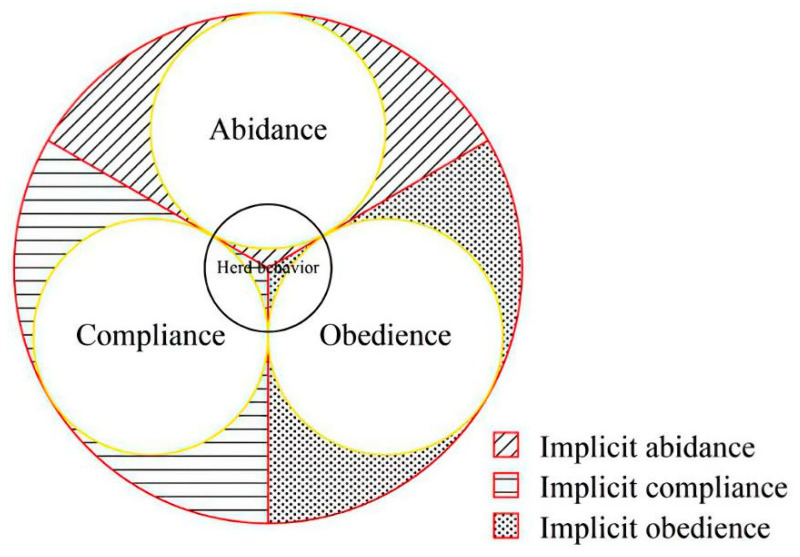
Types of conformity and their relationship.

**Figure 2:**
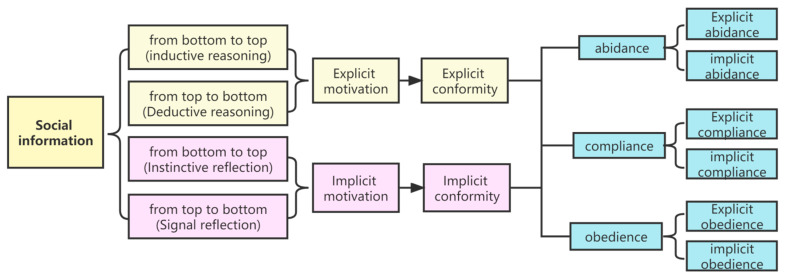
Processes involved in Information processing and types of internal attribution.

In fact, in addition to the individual’s emotional experience, implicit motivation can originate from the transfer of knowledge, drive for interest, etc. Extrinsic motivation in combination with social incentives influences behavior [37]. On this basis, we refer to automated conformity which is formed on the basis of simple perceptual organization (instincts or signal conditioning) as implicit conformity, and to conformity formed through complex mental processing as explicit conformity. Both implicit and explicit conformity can be divided into three types: abidance, compliance, and obedience, with each type having two kinds of expression: implicit and explicit. Therefore, conformity can be explicit and implicit; herd behavior (or cluster) is a particular form of conformity. Figure 1 shows the types of conformity and the relationship between them. Figure 2 shows the psychological processes of conformity information processing and forms of internal attribution.

The large circle represents conformity, and the three medium-sized circles inside the digger circle represent abidance, compliance, and obedience. The small circle passing through the tangent point of the inscribed circle represents conformity in a narrow sense, (i.e., herd behavior). Herd behavior, in its narrow sense refers to conformity. The shaded areas in the figure represent implicit conformity (including three types: implicit abidance, implicit compliance, and implicit obedience).

The social information pertaining to conformity is derived from the incentives, which is influenced by both internal and external information from family, social relations, environment, or educational policy. The internal incentive has a significant effect on all types of conformity. The environment incentive significantly affects abidance, family and social relation incentive affect compliance, and policy incentive substantially impacts obedience [17].

**Proposition** **3.** *SRC formation is influenced by many factors, such as people’s internal incentive, family incentive, social relations incentive, environment incentive, and educational policy incentive.*

### 2.2. Research Hypotheses

#### 2.2.1. Internal Incentives

Scientific research motivation is a kind of psychological motivation, which can drive researchers to participate actively in scientific research [39]. It can activate, maintain, and regulate scientific research behavior [40]. Research motivation is the psychological motivation that influences scholars to participate in scientific research [41]. It stems from scholars’ own scientific research interests, scientific research ideals, and social responsibilities [42]. Therefore, internal incentives may have a significant impact on teachers’ cognition.

**Hypothesis** **3A1.** *Internal incentives have a significant effect on scientific research abidance.*

**Hypothesis** **3A2.** *Internal incentives have a significant effect on scientific research obedience.*

**Hypothesis** **3A3.** *Internal incentives have a significant effect on scientific research compliance.*

#### 2.2.2. Family Incentives

Family incentives refer to the influence of the family atmosphere and family relationships on scientific research abidance. Family motivation is also an essential factor that affects the attitude of university teachers toward scientific research [43]. Families value emotion more, and hence the effect on compliance to scientific research is more pronounced [44], but the impact on other types of SRC may not be significant.

**Hypothesis** **3B1.** *The effect of family incentives on scientific research abidance is insignificant.*

**Hypothesis** **3B2.** *The effect of family incentives on scientific research obedience is insignificant.*

**Hypothesis** **3B3.** *Family incentives have a significant effect on scientific research compliance.*

#### 2.2.3. Social Relationship Incentives

Different individuals tend to form social relations through participation in social activities. This is no exception to scientific research [45]. When there is familiarity in researchers’ relationships, there is greater scientific research collaboration among them [46]. Therefore, the maintenance of social relationship incentives mainly depends on emotions. Emotions have a significant impact on scientific research compliance, but the impact on other types of SRC may not be significant.

**Hypothesis** **3C1.** *The effect of social relationship incentives on scientific research abidance is insignificant.*

**Hypothesis** **3C2.** *The effect of social relationship incentives on scientific research obedience is insignificant.*

**Hypothesis** **3C3.** *Social relationship incentives have a significant effect on scientific research compliance.*

#### 2.2.4. Environment Incentives

The incentives pertaining to scientific research environment will affect the researcher’s scientific conformity behavior. An excellent scientific research environment can enhance the scientific research output of scholars [47]. Existence of a healthy research atmosphere is likely to create an efficient working environment [48]. However, research culture and atmosphere are the product of individual cognitive processing. Therefore, environmental incentives are likely to have a significant impact on scientific research abidance. At the same time, the environment incentive effect on scientific research compliance and scientific research obedience may not be significant.

**Hypothesis** **3D1.** *Environment incentives have a significant effect on scientific research abidance.*

**Hypothesis** **3D2.** *The effect of environment incentives on scientific research obedience is insignificant.*

**Hypothesis** **3D3.** *The effect of environment incentives on scientific research compliance is insignificant.*

#### 2.2.5. Policy Incentives

Assessment criteria, promotion system, and research policy all affect teachers’ research motivation. Appropriate rewards increase motivation toward attainment of degree or completion of a course of study, research productivity, and impact [49]. Some scholars believe that introducing new funding policies are likely to enhance the efficiency of scientific research [50]. Policy making is motivating and will directly affect the interests of teachers. Therefore, policy incentives significantly impact scientific research obedience but have no significant impact on other types of SRC.

**Hypothesis** **3E1.** *Policy incentives have a significant effect on scientific research obedience.*

**Hypothesis** **3E2.** *Policy incentives have no significant effect on scientific research abidance.*

**Hypothesis** **3E3.** *Policy incentives have no significant effect on scientific research compliance.*

## 3. Study 1

### 3.1. Participants and Methods

#### 3.1.1. In-Depth Interview

We selected 15 participants including eight males and seven females; three of the participants were over 50 years old, seven were in the age range 40 to 49 years, and five of them were between 30 and 39 years. The participants comprised five professors, five associate professors, and five lecturers. They were selected using purposive sampling from seven universities, including Northeastern University, Liaoning University, Shenyang Normal University, Shenyang University of Technology, Shenyang Sport University, Shenyang Aerospace University, and Shenyang Jianzhu University. The faculty members who participated in the interviews were from general universities and “double-first-class” universities, and worked in the following colleges: School of Metallurgy, School of Business Administration, School of Life Science and Health, School of Music, School of Marxism, the School of Educational Sciences, School of Foreign Languages, School of Liberal Arts, School of Economics, School of Resources and Civil Engineering, School of Philosophy, School of Management, School of International Political Science, School of Mechanical Engineering and Automation, and School of Architecture and Planning, with a relatively balanced ratio.

A semi-structured interview was conducted and the following information was sought from the participants:

Please share your scientific research experience. The questions asked were: What is your motivation for scientific research? What is the incentive for scientific research?

#### 3.1.2. Research Procedure 

**Figure 3:**
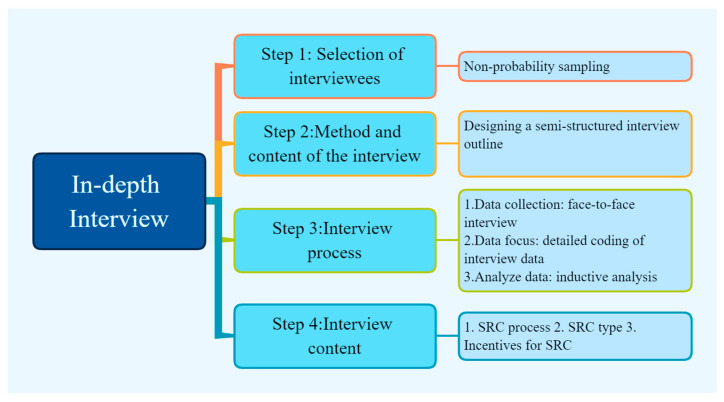
Interview procedure.

Study1 was a qualitative study, and the four steps of the interview are shown in Figure 3 below: selection of interviewees, the method and content of the interview, the interview process, and the content of the interview. The types of SRC and the associated incentives were determined on the basis of in-depth interviews for empirical analysis and confirmation of hypotheses. The details of the in-depth interview process are illustrated in Figure 3 below along with the research procedure.

### 3.2. Results

#### 3.2.1. SRC Process

According to the question of university teachers’ experience in scientific research work, their scientific research behavior is shaped by their cognition, experience, and pursuit of a scientific research connotation, scientific research product, or scientific research achievements. For example, Teacher X said: *“It is not only the routine work of teachers to engage in scientific research, but also a sense of responsibility. Scientific research is to conduct in-depth research in a specific field, which may be boring, but teachers also need to have a strong willpower to overcome challenges, only in this way will we meet our goal”*. Teacher Z said: *“When I first began working in scientific research institutes, I felt pleased with my ability to complete the assigned tasks. I gradually realized that teachers could not be separated from scientific research, irrespective of their job responsibilities. They also have to make students participate in competitions and apply for projects in addition to teaching them. I gradually realized that this kind of life, despite being busy, was still quite satisfying”*. Teacher M said: *“The purpose of scientific research is to develop and innovate based on the knowledge of predecessors, and to form its unique view by creating its research field. As a member of the college’s scientific research team, I am often proud to bring honor to the college and the school by contributing to various academic exchanges”.*

The teachers’ responses to the interview questions revealed the following: Teacher X viewed scientific research as a kind of consciousness and responsibility; Teacher Z viewed it as a tool for teaching and believed that it can bring him achievement and honor. Teacher M believed that scientific research not only aids achievement but also brings honor. Despite the differences in motivation and purpose, their scientific research interests were the same. As a concept, we call this external consistency of university teachers’ scientific research behavior influenced by internal and external factors as SRC.

#### 3.2.2. SRC Type

The answer to the question about the motivation of university teachers regarding scientific research reveals that the motivation is mainly cognitive, emotional, utilitarian, or unconscious motivation. There are four types of conformity: abidance, compliance, obedience, and unconscious conformity. For example, Teacher Y said: *“I engage in deep research in order to break past all kinds of superficial and even misleading fog, to grasp the international situation and development direction correctly so that the country and the people can make the right choice”*. Teacher T: *“ Primarily I conduct scientific research to pass the final examination at the end of the year. I pursue scientific research, as I do not want to be the last one in the final exam because as per the standards of the school the papers and projects should not only be characterized by quantity but also quality”.* Teacher W said: *“Pursuance of scientific research can enhance one’s self-esteem to some extent, and one is likely to feel satisfied after accomplishing something. Additionally, those engaged in scientific research have expectations from their families, since a dedicated program may reduce family time, but family members still support them and encourage them to work hard, so they are motivated tone more productive, and share the results their efforts with their family members”.* Teacher X said: *“I have been involved in scientific research for nearly twenty years. At first, I was confused. I didn’t know what exactly I was interested in. As I have achieved to a certain extent, I have found that scientific research, with its own research directions, also provides us with unique insights and ideas”*. To sum up, the 15 surveyed teachers’ SRC types can be divided into four categories among which, 46.67% accounts for scientific research abidance, 33.33% for scientific research compliance, 40.00% for scientific research obedience, and 33.33% for unconscious SRC (Supplementary Table S1). Since each individual had more than just one preference choice, the total score exceeds 100%.

#### 3.2.3. Incentives for SRC

With respect to incentives that are being provided to university teachers to engage in scientific research, the incentives university teachers pursue scientific research due to the following: internal incentives, family incentives, social relation incentives, environmental incentives, and policy incentives. For example, Teacher G said: *“For conducting scientific research, it is vital that you understand what you are trying to do, to know how you want to do it, to have a clear goal, and to keep your interests alive. There are bound difficulties when conducting research, such as experiments that require repeated attempts, corrections, and adjustments, and cannot be sustained without a strong interest and determination”.* Teachers D and H remarked: *“My college’s academic achievements and leadership had a greater impact on me. Of course, the leadership has its own expectations, but the administrators also hope to do well in scientific research to meet the leadership’s standards, and looking back at their past achievements can also be a motivating factor. Early in my career, I have been privileged to be exposed to many outstanding academicians. College leaders and older teachers have helped me a lot with my scientific research. They have also saved me from a number of detours along the way. Throughout the years, I have also improved myself in various aspects, so I am very grateful to them”*. Teacher G.Y. said: *“Colleges and universities should create a ‘positive, harmonious, and pragmatic’ scientific research environment for teachers in order to meet the needs of economic development. Such an environment contributes to the smooth progress of scientific research”*. Teacher W.D. said: *“Even though it’s important to have the support of your family and live up to their expectations, you also want to be promoted through research so that you can apply for more projects and have enough research funds to support your research*”. Teacher T commented: *“The level of the research team or the way they communicate has a great impact on my research. Research team members are capable of coming up with new ideas for solving problems and providing insights”*. The incentives for SRC of the 15 surveyed teachers were divided into five types: internal incentives accounted for 40%, family incentives for 26.67%, social relationship incentives for 26.67%, environmental incentives for 26.67%, and policy incentives for 26.67% (Supplementary Table S2). Since each individual had more than just one preference choice, the total score exceeds 100%.

## 4. Study 2

### 4.1. Research Method 

#### 4.1.1. Research Sampling Method

Three-stage stratified sampling method was used in this study. In the first stage, undergraduate universities in Shenyang were stratified into “double-first-class” universities and “ordinary universities”. The total number of teachers was 20,706 which included 4024 full-time teachers from “double-first-class” universities and 16,682 full-time teachers from ordinary universities. Out of the 360 samples, 69 samples were taken from “double-first-class” universities and 291 samples were taken from ordinary universities. In the second stage, they were classified according to the disciplines: humanities and social sciences, science and technology, agricultural sciences, medicine and science, arts, and sports. Simple random sampling was used to draw a list of universities and the sample size was determined on the basis of the proportion of the full-time teachers. In the third stage, full-time faculty members were selected through convenience sampling from the selected undergraduate universities. The sample distribution has been illustrated in Table 1.

#### 4.1.2. Research Tools and Procedure

Firstly, a questionnaire was designed on the basis of the responses obtained in the extensive interviews of the college teachers to seek information regarding their scientific research behavior and results. The final survey questionnaire included 12 items that pertained to basic personal information, 22 items pertaining to SRC types, and 18 items pertaining to SRC incentives. The questionnaire is a five-point Likert scale, with scores on responses ranging from 1 to 5 (“1”= least consistent; and “5” = most consistent).

The second step was to collect data. These teachers were selected from seven universities in Shenyang, a sample of universities was divided into “double-first-class” universities and ordinary universities. In the second stage, the sampling subjects were divided into five groups.

In the third stage, college teachers who taught different subjects were selected through random sampling. A total of 360 questionnaires were distributed and 349 questionnaires were considered for data analysis; the remaining 11 were invalid because of incomplete or missing answers. The data collection procedure was conducted from 5 September 2018, to 20 October 2018. The effective rate was 96.9%. Data collection was followed by exploratory factor analysis to explore whether the quantitative data can be condensed into several aspects (factors), and each aspect (factor) corresponds to the item.

On the one hand, the Kaiser–Meyer–Olkin (KMO) values of the two scales were higher than 0.8, indicating that it is very suitable for factor analysis. On the other hand, after the Bartlett test of the two scales, the corresponding *p*-values were both less than 0.05, which also indicates that factor analysis was suitable. Based on the results of the factor analysis, three factors were extracted from the SRC-type questionnaire, and five factors were extracted from the SRC incentives questionnaire. This was followed by confirmatory factor analysis. The average variance extracted (AVE) and construct reliability (CR) values were judged separately for convergent validity analysis based on the extracted common factors. Some sample items included in the SRC scale are depicted in Table 2.

Finally structural equation modeling was carried out using the AMOS software. The SRC incentives corresponding to the three SRC types were summarized to create a structural equation path.

### 4.2. Results

#### 4.2.1. Analysis of SRC Types

Statistical analyses of the types of SRC show that these types can be categorized into three categories: abidance, compliance, and obedience. The average scores of 349 college teachers’ for scientific research abidance, scientific research obedience, and for scientific research compliance were 3.8813, 4.0100, and 3.7563, respectively. Scientific research obedience had the highest mean value. With respect to types of conformity, the results revealed 45.6% accounted for scientific research obedience, 32.4% for scientific research abidance, and 22.0% for scientific research compliance. Results of factor analysis revealed that KMO was 0.875, indicating that the questionnaire was suitable for exploratory factor analysis.

Exploratory factor analysis was used to extract the common factors from the SRC questionnaire (Table 3). Three factors with eigenvalues greater than one were identified as standard and contributed to 53.803% of the variance. According to the scree plot, the number of factors corresponding to the discount from steep to stable is the number of reference extraction factors, so three factors in the SRC type were confirmed (Supplementary Figure S1). The three common factors are abidance, compliance, and obedience. The confirmatory factor analysis of SRC by AMOS23.0 showed that the standard factor load of each item was more significant than 0.5, the composite reliability (CR) was more significant than 0.7, and the average variance extracted (AVE) value was more significant than 0.4 (Supplementary Tables S3–S5). Table 4 shows that this dimension has high convergence validity.

#### 4.2.2. Analysis of SRC Incentives

On the basis of the responses on the questionnaire on the incentives for SRC, five aspects were identified: internal incentives, family incentives, social relationship incentives, environmental incentives, and policy incentives. The 349 college teachers scored an average of 3.98 on internal incentives, 3.75 on family incentives, 3.61 on social relationship incentives, 4.05 on environment incentives, and 4.16 on policy incentives. Factor analysis revealed that KMO was 0.828, indicating that the questionnaire was suitable for exploratory factor analysis.

The common factors were extracted from the SRC questionnaire. The results indicate that five common factors were extracted with eigenvalues greater than one, with a cumulative variance contribution of 60.328%. Table 5 shows that the factor loads of each item of the rotated component matrix meet the standard of 0.5. According to the scree plot, the number of factors corresponding to the discount from steep to stable is the number of reference extraction factors, so there are five factors in the SRC incentives (Supplementary Figure S2). Based on the results pertaining to SRC incentives, there are five public factors: internal incentive, family incentive, social relationship incentive, environmental incentive, and policy incentive. According to the confirmatory factor analysis model, the AVE and CR results are shown in Supplementary Tables S6–S10. Table 6 shows that this dimension has high convergence validity.

#### 4.2.3. Reliability Analysis of the Scale

The results of the split half-reliability analysis show that the correlation between the scores on the two tests is 0.654, and the reliability coefficients for each part are 0.882 and 0.839, respectively, indicating that the scale has excellent internal reliability. The total Cronbach’s alpha coefficient of the questionnaire is 0.914, which indicates that the questionnaire is highly reliable (Supplementary Table S11).

All types of SRC type questionnaires have reliability higher than 0.8, and the total Cronbach’s alpha coefficient of the questionnaire is 0.887, which indicates that the SRC type questionnaire is very reliable.

According to the SRC incentives questionnaire, the reliability of all inducing factors was higher than 0.7. The total factor Cronbach’s alpha coefficient of inducing factors was 0.829, indicating that the reliability of SRC incentives questionnaire was outstanding.

#### 4.2.4. Validity Analysis of the Questionnaire

SRC type questionnaires are based on the internal attribution hypothesis of conformity. According to the nature of conformity motivation, items fall into three categories: cognitive, emotional, and utilitarian. In addition, the items of the incentive questionnaire were formulated on the basis of in-depth interviews, which were based on SRC.

The number of scientific research achievements of university teachers per year showed a significant correlation between the types of SRC (*p* < 0.01). Scientific research abidance (t = 0.68, *p* < 0.01), scientific research compliance (t = 0.41, *p* < 0.01), and scientific research obedience (t = 0.43, *p* < 0.01) are positively correlated with scientific research results (Supplementary Table S12).

The exploratory and confirmatory factor analyses revealed that the questionnaire had good construct validity, that is, there was agreement between the results obtained by the questionnaire and the theory on which it was based.

#### 4.2.5. Path Analysis of SRC Model 

An exploratory structural equation model was used to examine the types of SRC and SRC incentives among university teachers, revealing the influence of the path analysis. Table 7 gives the results of model fitting indexes, which show that the model fits well.

**Figure 4:**
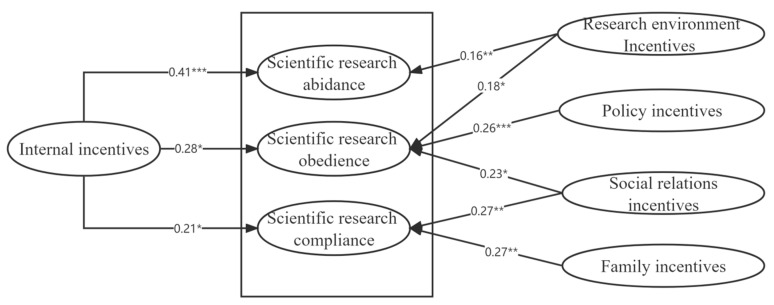
Results of model path analysis. (* *p* < 0.01, ** *p* < 0.05,*** *p* < 0.001).

Based on structural equation model analysis through AMOS23.0, Figure 4 illustrates the analysis of model path. It is necessary to mention that we removed the insignificant paths from the path diagram and only 9 significant paths have been represented. The arrows represent the relationship between the direct positive effects of the variables and the standardized path coefficients. The direct output of AMOS is illustrated in Supplementary Figure S3.

The results of the model path analysis are presented in Table 8. Furthermore, 13 of the 15 research hypotheses within the model have been validated. Most of them validate our proposed hypothesis and are partially verified by the theory. In the relationship hypothesis, social relationships and the environment did not significantly influence scientific research obedience. However, the actual data analysis showed that they both significantly influenced scientific research obedience (*p* < 0.05). The internal incentives mainly affected the abidance (*p* < 0.001), but also had a significant effect on compliance and obedience (*p* < 0.05). The environment incentives mainly affected abidance (*p* < 0.01), but also had a significant effect on obedience (*p* < 0.05). Family and social relationships incentives had a significant effect on compliance (*p* < 0.01), social relationship incentives had a significant effect on obedience (*p* < 0.05), and policy incentives had a significant effect on obedience (*p* < 0.001).

The results show that the model can support the hypothesis of scientific research of university teachers except in some individual cases. Only Hypothesis 3C2 and Hypothesis 3D2 were rejected, whereas the rest of the hypotheses were accepted.

## 5. Discussion and Conclusion

Based on the scientific research behavior of university teachers, this study obtained the following conclusions through in-depth interviews with 15 subjects and a questionnaire survey of 349 subjects.

### 5.1. Propose the Information Processing Patterns for SRC

The results of in-depth interviews indicate that research behavior depends on the input of social information, which may be internal or external. Internal information is acquired from the knowledge and experience that individuals accumulate during their study, work, and life, as well as from their habits and innate instincts; external information comes from the environment in which the individuals work, study and live, that includes family, school, society, etc. [35]. Social information constitutes the ‘motivation of research behavior, and when a person displays research behavior, it proves that they accept the influence of relevant information and hence display behavior that is reflective of the influence of social information, which we call SRC. An individual acts as a source of various kinds of internal and external information; the social information needs to be processed and filtered in the process of establishing research compliance [32]. The processing of social information may be perceptual (unconscious), such as compliance formed by habit or instinct, or rational (conscious), such as conformity formed by complex reasoning and judgment. The former can be referred to as implicit conformity, and the latter as epiphenomenal conformity. Both implicit and explicit conformity are motivated behaviors, with implicit conformity corresponding to implicit motivation and explicit conformity corresponding to explicit motivation.

### 5.2. Propose the Internal Attribution Property and Motivation Classification of SRC

A traditional view of external attribution defines conformity as behavior due to the influence of the majority [12], compliance as behavior due to others’ demands, and obedience as behavior due to others’ orders. However, the findings of this study are contradictory [51]. In this study, conformity is considered to be divided into abidance, obedience, and compliance. The internal and external factors are combined as per the conformity view. Conformity arises when social information is influenced by incentives [52]. Conformity behavior consists of a motivational strategy formed through human information processing. Conformity may have cognitive, emotional, or utilitarian motivations. Because there are external cases of conformity behavior, including external abidance, obedience and compliance, and there is also an implicit level of unconscious conformity, which is not easy to measure, hence the main study explores external conformity. Hence, conformity can be divided into four types: abidance, compliance, obedience, and unconscious conformity. Quantitative research on conformity concluded that conformity includes three behaviors: abidance, compliance, and obedience. These behaviors are divided into implicit and explicit conformity. This classification refines the nature of internal attribution.

The factor analysis of the SRC questionnaires of 349 teachers showed three types of motivation: cognitive motivation, affective motivation, and utilitarian motivation. Interviews revealed the distribution of unconscious research motivation items. Different types of motivations for conformity exist, for example “being engaged in scientific research is a natural response” and is a part of scientific research abidance. Moreover, “seeing others engaged in scientific research will make me follow the scientific research” as part of scientific research compliance. It shows that the unconscious scientific research behavior referred to in the hypothesis formulated for the research is also a kind of motivation [53]. However, this kind of motivation is implicit, and the conformity behavior it produces is also implicit (i.e., implicit abidance, implicit compliance, and implicit obedience).

### 5.3. Propose Five Incentives for SRC

Although there are many studies on SRC incentives [54], from the perspective of internal attribution of conformity, there are few articles on the research conformity behavior and incentives of university teachers. Factor analysis revealed five types of incentives that influence the SRC of university teachers: internal incentives, family incentives, social relationship incentives, environment incentives, and policy incentives.

The internal incentives mainly emerge from human experiences, habit or instinct, and their information may be cognitive, emotional, or utilitarian. Therefore, all types of conformity are affected by internal incentives. The environmental incentive pertaining to scientific research mainly affects the abidance behavior, the family incentive and the social relation incentive mainly affect the compliance behavior, and the policy incentive mainly affects the obedience behavior [55]. Family is a human emotional support and an essential guarantee to maintain the research motivation of university teachers. Research shows that family incentives have a significant effect on research compliance (*p* < 0.01), i.e., a warm family atmosphere and harmonious family relationships influence college teachers’ emotion-based motivation and promote their research compliance, when teachers’ research behaviors and their achievements will become a tool to pleasure family members and also reverse motivate teachers’ research [43]. Social interactions between individuals form human social relationship incentives. According to Maslow, interaction is a basic human need, and the basis that can sustain interaction is emotion or benefit [45]. Studies have shown that social relationship incentives have a significant effect on both research compliance (*p* < 0.01) and obedience (*p* < 0.05). The mechanism by which social relationship inducements affect research compliance is motivated by the expectation of research compliance subjects to establish and maintain good social relationship inducements because of the emotional experiences that can be gained through research. Of course, social relationship incentives may also serve as a bond of interest, earning the respect of others or leadership accolades through research obedience. Environmental determinists believe that the environment plays a decisive role on human behavior [47]. Research environment incentives include both hardware and software, i.e., hardware refers to research equipment and facilities, while software refers to the cultural atmosphere of research. Studies have shown that research environmental incentives have a significant effect on research abidance (*p* < 0.01) and obedience (*p* < 0.05). Obviously, when a university faculty member is in a group with a strong research environment, it will naturally increase the recognition of research and improve research abidance. When a faculty member sees that others have been promoted to higher titles or have increased their visibility due to research, it also promotes the formation of his or her research obedience. The development of research policies in higher education institutions is often guided by the research interests of teachers, and interests are the incentives for teachers’ research, and these incentives may be expressed in the form of research awards and job promotion [50]. The study showed that there was a highly significant effect of policy incentives on research obedience (*p* < 0.001). In contrast, policy incentives had no significant effect on other types of SRC.

To summarize, during the interview process, the teachers interviewed mentioned that establishment of social relationships, mainly with the members of the research team and leaders, etc., is likely to influence their own research. When they face any confusion with respect to their research work, the members of the research team can provide new ideas or can pool in ideas; hence, this is conducive to finding solutions to research problems [56]. The research team can be strengthened and a congenial humanistic environment that promotes scientific research can be created, and improvement in quality of research can be sought by the team [57].

According to the two-factor theory, research conditions being a health care factor contributes to the retention of university teachers’ enthusiasm in research. Perfect research equipment enable teachers to engage in research activities under congenial conditions that facilitate progression of research at a fast pace; this helps the university teachers to engage in academic and technologically innovative research, etc. [15]. Only by making great efforts to build a good research environment can the universities guarantee teachers’ research enthusiasm while motivating them to make significant, innovative achievements and maintain university teachers’ SRC behavior.

## 6. Limitations and Recommendations for Future Research

Despite the academic contribution of this study, the following limitations exist: firstly, the study was conducted with college teachers in Shenyang, Liaoning Province, and the sampling range was limited to 7 of the 19 undergraduate universities in Shenyang. The data reflected can only illustrate the SRC types and motivational characteristics of college teachers in Shenyang, Liaoning Province, and the analysis of SRC types and motivational models of college teachers nationwide needs further research. Meanwhile, the sample size of this survey is 360, which meets the statistical sample size requirement, but the sample size must be increased in the future to make the study more general. Hence, the accuracy of classification of conformity motivation is likely to be affected. Future research will expand the scope of the survey. Secondly, the 3C2 and 3D2 hypotheses were rejected, as the model path analysis showed that the environment and social relationship incentives significantly affected obedience. Perhaps this is because the university teachers are in an environment or social relations full of competing interests, hence, further validation is required in the future. The psychology of research conformity is studied in the context of traditional Chinese culture, and some studies have shown that Chinese researchers are more emotionally motivated to be compliant [58]. Finally, the qualitative study mainly used in-depth interviews with the aim of analyzing the types and motivational characteristics of SRC, but there are limitations, as the unstructured nature of the survey makes the results very susceptible to the interviewer’s own influence, and the integrity of the quality of the results is very dependent on the interviewer’s skill, which may lead to one-sided or superficial information obtained in the study.

The following aspects can be considered in future studies.

Firstly, in order to have a more comprehensive understanding of the types and triggering factors of SRC among college teachers, the scope and capacity of the sample should be increased in future studies, which can make the research results more rigorous and scientific.

Secondly, future research can use various qualitative research methods, in addition to in-depth interviews, case studies, and narrative studies, to understand the dynamics of college teachers’ scientific research from an all-round and multi-dimensional perspective, and explore the law of conformity from different perspectives.

Thirdly, future studies exploring SRC with an international perspective or comparative studies could be conducted to enrich the sample selection of SRC studies.

## Figures and Tables

**Table 1:** Sample distribution of university.

University Name	University Type	“Double-First-Class” University	Sampling Size
Northeastern University	Comprehensive	Yes	46
Liaoning University	Comprehensive	Yes	23
Shenyang Normal University	Normal	No	79
Shenyang University of Architecture	Engineering	No	82
China Medical University	Medicine	No	60
Shenyang Agricultural University	Agriculture	No	46
Shenyang Institute of Sports	Sports	No	24
Total			360

**Table 2:** Description and sample items of the SRC scale.

Scale	Scale Dimension Items	Description and Sample Items
SRC scale	Scientific research abidance	I always find scientific research in the university to be enjoyable.
	Scientific research compliance	I was expected to do scientific research by my family or friends and did not want to disappoint them.
	Scientific research obedience	I conduct scientific research to get promoted or to receive awards.

**Table 3:** Explanation of total variance.

Element	% of Variance (Unrotated)			% of Variance (Rotated)		
	Eigen Value	Variance%	Accumulation %	Eigen Value	Variance%	Accumulation %
1. Scientific research abidance	6.745	30.658	30.658	5.084	23.108	23.108
2. Scientific research compliance	2.637	11.987	42.645	3.687	16.759	39.867
3. Scientific research obedience	2.455	11.158	53.803	3.066	13.935	53.803

**Table 4:** Indicators of SRC type model fit.

Indicators	CMIN	DF	CMIN/DF	GFI	IFI	TLI	CFI	RMSEA
Numerical value	458.358	205	2.236	0.900	0.919	0.908	0.919	0.06

**Table 5:** Explanation of total variance.

Element	% of Variance (Unrotated)			% of Variance (Rotated)		
	Eigen Value	Variance%	Accumulation %	Eigen Value	Variance%	Accumulation %
1. Internal incentive	4.679	25.993	25.993	2.472	13.734	13.734
2. Family incentive	2.037	11.318	37.310	2.401	13.339	27.073
3. Social relationship incentive	1.595	8.859	46.169	2.171	12.060	39.132
4. Environmental incentive	1.445	8.031	54.200	1.984	11.024	50.157
5. Policy incentive	1.103	6.128	60.328	1.831	10.171	60.328

**Table 6:** Indicators of SRC incentive type model fit.

Indicators	CMIN	DF	CMIN/DF	GFI	IFI	TLI	CFI	RMSEA
Numerical value	187.262	125	1.498	0.944	0.962	0.953	0.962	0.038

**Table 7:** Model fitting indicators.

Indicators	CMIN	DF	CMIN/DF	GFI	IFI	TLI	CFI	RMSEA
Numerical value	1154.177	714	1.616	0.906	0.915	0.865	0.914	0.042

**Table 8:** Analysis of path coefficients.

Analysis Path	Variable Relation	Path Coefficient	S.E.	C.R.	*p*	Verification Results
3A1	Internal incentives → scientific research abidance	0.409	0.087	4.706	***	Established
3A2	Internal incentives → scientific research obedience	0.282	0.118	2.392	*	Established
3A3	Internal incentives → scientific research compliance	0.206	0.091	2.253	*	Established
3B1	Family incentives → scientific research abidance	0.070	0.070	1.001	0.317	Established
3B2	Family incentives → scientific research obedience	−0.060	0.111	−0.540	0.589	Established
3B3	Family incentives → scientific research compliance	0.274	0.090	3.046	**	Established
3C1	Social relations incentives → scientific research abidance	0.013	0.065	0.195	0.845	Established
3C2	Social relations incentives → scientific research obedience	0.229	0.105	2.182	*	invalid
3C3	Social relations incentives → scientific research compliance	0.266	0.084	3.156	**	Established
3D1	Environment incentives → scientific research abidance	0.157	0.058	2.698	**	Established
3D2	Environment incentives → scientific research obedience	0.176	0.090	1.967	*	invalid
3D3	Environment incentives → scientific research compliance	0.001	0.069	0.009	0.992	Established
3E1	Policy incentives → scientific research obedience	0.260	0.066	3.965	***	Established
3E2	Policy incentives → scientific research abidance	−0.063	0.040	−1.573	0.116	Established
3E3	Policy incentives → scientific research compliance	−0.017	0.049	−0.341	0.733	Established

Note: * significant at *p* < 0.05 level; ** significant at the *p* < 0.01 level; *** significant at *p* < 0.001 level.

## Data Availability

The raw data supporting the conclusions of this article will be made available by the authors without undue reservation.

## Supplementary Materials

The following supporting information can be downloaded at: https://www.mdpi.com/article/10.3390/brainsci12101302/s1, Table S1: The SRC types of the teachers interviewed; Table S2: The SRC incentives of the teachers interviewed; Figure S1: SRC type scree plot; Figure S2: SRC incentives scree plot; Table S3: Confirmatory factor analysis results of scientific research abidance; Table S4: Confirmatory factor analysis results for scientific research obedience; Table S5: Confirmatory factor analysis results of scientific research compliance; Table S6: Results of confirmatory factor analysis of internal incentives; Table S7: Results of confirmatory factor analysis of family incentives; Table S8: Results of confirmatory factor analysis of social relations incentives; Table S9: Results of confirmatory factor analysis of environmental incentives; Table S10: Results of confirmatory factor analysis of policy incentives; Table S11: Analysis of the half-reliability of the scientific research conformity scale; Table S12: Criteria for classification and classification of papers; Figure S3: The direct output of AMOS.

## References

[1] Adam S., Raphael D.D., Macfie A.L. (1759). The Theory of Moral Sentiments.

[2] Gustave L.B. (1895). The Crowd: A Study of the Popular Mind.

[3] Thorstein V. (1899). The Theory of the Leisure Class: An Economic Study of Institutions.

[4] Gabriel T. (1903). The Laws of Imitation.

[5] Goncalo J.A., Duguid M.M. (2012). Follow the crowd in a new direction: When conformity pressure facilitates group creativity (and when it does not). Organ. Behav. Hum. Decis. Process..

[6] Cialdini R.B., Goldstein N.J. (2004). Social influence: Compliance and conformity. Annu. Rev. Psychol..

[7] Wice M., Davidai S. (2020). Benevolent Conformity: The Influence of Perceived Motives on Judgments of Conformity. Personal. Soc. Psychol. Bull..

[8] Keuschnigg M. (2012). Conformity through herd behavior—Theoretical arguments and empirical results on the emergence of bestsellers. Koln. Z. Fur Soziologie Und Soz..

[9] Biderman D., Shir Y., Mudrik L. (2020). B or 13? Unconscious Top-Down Contextual Effects at the Categorical but Not the Lexical Level. Psychol. Sci..

[10] Allport F.H. (1924). Social Psychology.

[11] Chen M. (2020). The research of human individual’s conformity behavior in emergency situations. Libr. Hi Tech.

[12] Moscovici S., Lage E., Naffrechoux M. (1969). Influence of a consistent minority on the responses of a majority in a color perception task. Sociometry.

[13] Moscovici S. (1976). Social Influence and Social Change.

[14] Levine J.M., Hogg M.A., Abrams D. (2017). Factional conflict in groups: How majorities and minorities relate to one another. Group Process. Intergroup Relat..

[15] Song G., Ma Q., Wu F., Li L. (2012). The psychological explanation of conformity. Soc. Behav. Personal..

[16] Song G., Wang S., Wu J. The Psychological Explanation of Compliance. Proceedings of the 4th international conference on arts, design and contemporary education (icadce 2018).

[17] Fiedler K. Ethical norms and moral values among scientists Applying Conceptions of Morality to Scientific Rules and Practices. Proceedings of the 18th Sydney Symposium of Social Psychology, University of New South Wales.

[18] Li Y., Lu X., Zheng W., Luo J. (2021). The role of the mPFC in the social influence of majority and expert opinion. Neuropsychologia.

[19] Schnuerch R., Gibbons H. (2014). A Review of Neurocognitive Mechanisms of Social Conformity. Soc. Psychol..

[20] Chen X., Li S., Zhang Y., Zhai Y., Zhang Z., Feng C. (2022). Different drives of herding: An exploratory study of motivations underlying social conformity. PsyCh J..

[21] Horry R., Palmer M.A., Sexton M.L., Brewer N. (2012). Memory conformity for confidently recognized items: The power of social influence on memory reports. J. Exp. Soc. Psychol..

[22] Rochelle T.L., Yim K.H. (2015). Assessing the Factor Structure of the Chinese Conformity to Masculine Norms Inventory. J. Psychol..

[23] Sherif M. (1937). An experimental approach to the study of attitudes. Sociometry.

[24] Asch S.E. (1955). Opinions and social pressure. Sci. Am..

[25] Gueguen N., Marchand M., Pascual A., Lourel M. (2008). Foot-in-the-door technique using a courtship request: A field experiment. Psychol. Rep..

[26] Hollebeek L.D., Sprott D.E., Sigurdsson V., Clark M.K. (2022). Social influence and stakeholder engagement behavior conformity, compliance, and reactance. Psychol. Mark..

[27] Plohl N., Musil B. (2021). Modeling compliance with COVID-19 prevention guidelines: The critical role of trust in science. Psychol. Health Med..

[28] Xiao B., Song G. (2022). Association between Self-Efficacy and Learning Conformity among Chinese University Students: Differences by Gender. Sustainability.

[29] Kassin S., Fein S., Markus H.R. (2011). Social Psychology.

[30] Kelman H.C. (1958). Compliance, identification, and internalization three processes of attitude change. J. Confl. Resolut..

[31] Nail P.R., MacDonald G., Levy D.A. (2000). Proposal of a four-dimensional model of social response. Psychol. Bull..

[32] Xiao B., Song G. (2022). The Impact of Family Socioeconomic Status on Learning Conformity among Chinese University Students: Self-Efficacy as Mediating Factor. Sustainability.

[33] Song G. (1997). A New Understanding of Herd Behavior. Psychol. Sci..

[34] Song G. (2002). Recognition of Herd Behavior. Psychol. Sci..

[35] Gareau A., Gaudreau P. (2017). Working memory moderates the effect of the integrative process of implicit and explicit autonomous motivation on academic achievement. Br. J. Psychol..

[36] Gareau A., Gaudreau P., Boileau L. (2019). Past academic achievement contributes to university students’ autonomous motivation (AM) which is later moderated by implicit motivation and working memory: A Bayesian replication of the explicit-implicit model of AM. Learn. Individ. Differ..

[37] Resh W.G., Marvel J.D., Wen B. (2019). Implicit and Explicit Motivation Crowding in Prosocial Work. Public Perform. Manag. Rev..

[38] Slabbinck H., Van Witteloostuijn A. (2020). Explicit and Implicit Basic Human Motives, and Public Service Motivation. Front. Psychol..

[39] Ryan J.C. (2014). The work motivation of research scientists and its effect on research performance. R&D Manag..

[40] Green S.K., Lightfoot M.A., Bandy C., Buchanan D.R. (1985). A General Model of the Attribution Process. Basic Appl. Soc. Psychol..

[41] Ballesteros-Rodríguez J.L., De Saá-Pérez P., García-Carbonell N., Martín-Alcázar F., Sánchez-Gardey G. (2020). Exploring the determinants of scientific productivity: A proposed typology of researchers. J. Intellect. Cap..

[42] Berbegal-Mirabent J., Gil-Doménech D., Alegre I. (2018). Why Would You Ever Want to Become An Academic Entrepreneur?.

[43] Borrego Á., Barrios M., Villarroya A., Ollé C. (2010). Scientific output and impact of postdoctoral scientists: A gender perspective. Scientometrics.

[44] Rothausen-Vange T.J., Marler J.H., Wright P.M. (2005). Research Productivity, Gender, Family, and Tenure in Organization Science Careers. Sex Roles.

[45] Pepe A. (2011). The relationship between acquaintanceship and coauthorship in scientific collaboration networks. J. Am. Soc. Inf. Sci. Technol..

[46] Barabási A.L., Jeong H., Néda Z., Ravasz E., Schubert A., Vicsek T. (2002). Evolution of the social network of scientific collaborations. Phys. A Stat. Mech. Its Appl..

[47] Fox M.F., Nikivincze I. (2021). Being highly prolific in academic science: Characteristics of individuals and their departments. High. Educ..

[48] Torrisi B. (2013). Academic productivity correlated with well-being at work. Scientometrics.

[49] Graddy-Reed A., Lanahan L., D’Agostino J. (2021). Training across the academy: The impact of R&D funding on graduate students. Res. Policy.

[50] Kim W., Min S. (2020). The effects of funding policy change on the scientific performance of government research institutes. Asian J. Technol. Innov..

[51] Song G., Wang S. (2019). Process and attribution analysis of social conformity. Soc. Sci..

[52] Sun J.C.-Y., Syu Y.-R., Lin Y.-Y. (2017). Effects of conformity and learning anxiety on intrinsic and extrinsic motivation: The case of Facebook course groups. Univers. Access Inf. Soc..

[53] Bargh J.A., Schwader K.L., Hailey S.E., Dyer R.L., Boothby E.J. (2012). Automaticity in social-cognitive processes. Trends Cogn. Sci..

[54] Suo Q. (2016). Chinese Academic Assessment and Incentive System. Sci. Eng. Ethics.

[55] Leong C., Lebel L. (2020). Can conformity overcome the yuck factor? Explaining the choice for recycled drinking water. J. Clean. Prod..

[56] Lonnqvist J.E., Walkowitz G., Wichardt P., Lindeman M., Verkasalo M. (2009). The moderating effect of conformism values on the relations between other personal values, social norms, moral obligation, and single altruistic behaviours. Br. J. Soc. Psychol..

[57] Goette L., Tripodi E. (2021). Social Influence in Prosocial Behavior: Evidence from a Large-Scale Experiment. J. Eur. Econ. Assoc..

[58] Wang Z., Meltzoff A.N. (2020). Imitation in Chinese Preschool Children: Influence of Prior Self-Experience and Pedagogical Cues on the Imitation of Novel Acts in a Non-Western Culture. Front. Psychol..

