# Supplementary material for: Scientific Research Conformity of University Teachers: Role of Incentives and Internal Attribution
Source: Brain Sci. 2022 Sep 27;12(10):1302. doi: 10.3390/brainsci12101302 (PMC9599667; doi:10.3390/brainsci12101302)

Supplementary Table S1 *The SRC types of the teachers interviewed*

Types / Interviewees	Scientific research abidance	Scientific research compliance	Scientific research obedience	Unconscious Scientific research conformity
GX1	+			
DXJ2	+		+	+
DHX3		+		
PL4			+	
ZX5			+	+
ZY6	+			
TZ7			+	
MQL8		+		
WLJ9	+	+		
SJF10		+		+
GYL11	+			
XZH12	+			+
YT13	+			
WD14		+	+	
ZLP15			+	+

Note: “+” indicates the SRC type of the teachers interviewed

Supplementary Table S2 *The SRC incentives of the teachers interviewed*

Incentives / Interviewees	Internal incentives	Family incentives	Social relations incentives	Environment incentives	Policy incentives
GX1	+				
DXJ2	+				
DHX3			+	+	
PL4				+	+
ZX5		+			+
ZY6	+				+
TZ7			+		
MQL8		+		+	
WLJ9			+		
SJF10			+		
GYL11	+			+	
XZH12	+				
YT13	+	+			
WD14		+			+
ZLP15	+				

Note: “+” indicates the type of incentives of the teachers interviewed.

Supplementary Figure S1 *SRC type scree plot*

Note: When the fold line suddenly becomes smooth from steep, the number of factors corresponding to steep to smooth is the number of reference extracted factors. As can be seen from the figure, the eigenroot values of the principal components start to decline slowly from the fourth factor, and we can choose to

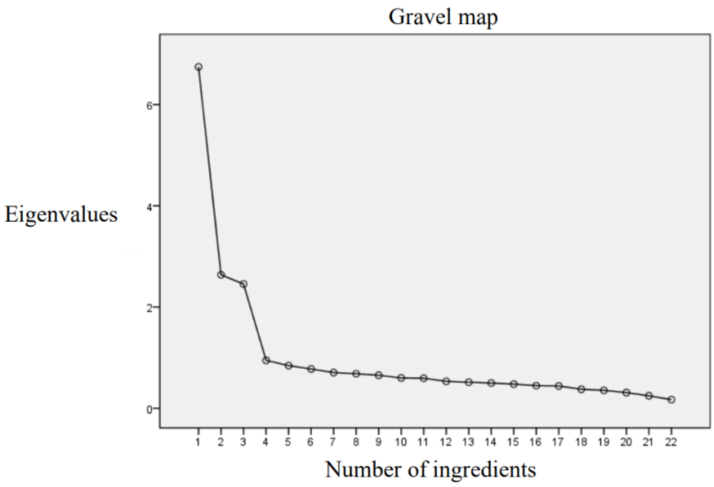

retain three factors when the contribution of the cumulative explanation of the factors meets 90%.

Supplementary Figure S2 *SRC* incentives *scree plot*

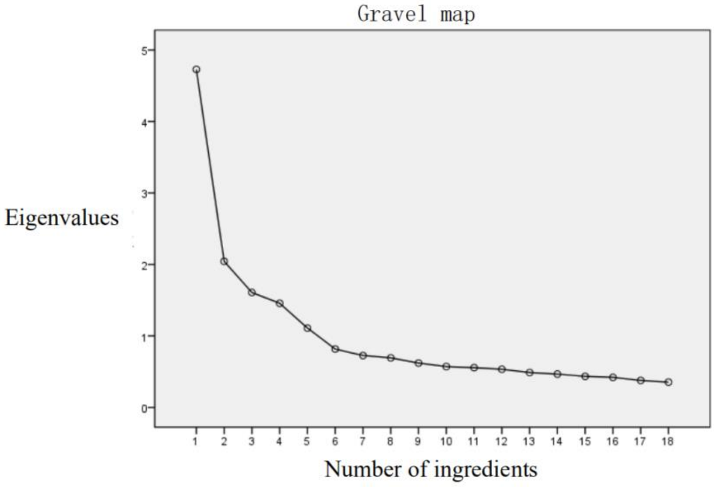

Note: When the fold line suddenly becomes smooth from steep, the number of factors corresponding to steep to smooth is the number of reference extracted factors. As can be seen from the figure, the eigenroot values of the principal components start to decline slowly from the sixth factor, and we can choose to retain five factors when the contribution of the cumulative explanation of the factors meets 90%.

Supplementary Table S3*Confirmatory factor analysis results of scientific research abidance*

Dimensions	Item	Normalized factor load	CR	AVE
Scientific research abidance	B1	0.605	0.8981	0.4711
	B2	0.564		
	B5	0.661		
	B7	0.772		
	B8	0.75		
	B9	0.729		
	B12	0.639		
	B13	0.745		
	B16	0.627		
	B17	0.737		

Supplementary Table S4 *Confirmatory factor analysis results for scientific research obedience*

Dimensions	Item	Normalized factor load	CR	AVE
Scientific research obedience	B3	0.78	0.8226	0.4849
	B4	0.799		
	B10	0.664		
	B19	0.632		
	B21	0.581		

Supplementary Table S5 *Confirmatory factor analysis results of scientific research compliance*

Dimensions	Item	Normalized factor load	CR	AVE
Scientific research compliance	B6	0.632	0.8426	0.4383
	B11	0.65		
	B14	0.848		
	B15	0.661		
	B18	0.69		
	B20	0.53		
	B22	0.577		

Supplementary Table S6 *Results of confirmatory factor analysis of internal incentives*

Dimensions	Item	Normalized factor load	CR	AVE
Internal incentives	C1	0.717	0.7451	0.4723
	C2	0.610		
	C3	0.644		
	C4	0.508		
	C5	0.550		

Supplementary Table S7 *Results of confirmatory factor analysis of family incentives*

Dimensions	Item	Normalized factor load	CR	AVE
Family incentives	C6	0.625	0.7104	0.4504
	C7	0.687		
	C8	0.699		

Supplementary Table S8 *Results of confirmatory factor analysis of social relations incentives*

Dimensions	Item	Normalized factor load	CR	AVE
Social relations incentives	C9	0.555	0.6772	0.4143
	C10	0.643		
	C11	0.722		

Supplementary Table S9 *Results of confirmatory factor analysis of environmental incentives*

Dimensions	Item	Normalized factor load	CR	AVE
Environmental incentives	C12	0.66	0.7915	0.4874
	C13	0.737		
	C14	0.681		
	C15	0.712		

Supplementary Table S10 *Results of confirmatory factor analysis of policy incentives*

Dimensions	Item	Normalized factor load	CR	AVE
Policy incentives	C16	0.669	0.7723	0.5317
	C17	0.730		
	C18	0.784		

Supplementary Table S11 *Analysis of the half-reliability of the scientific research conformity scale*

Reliability statistic			
**Cronbach's Alpha**	**Part 1**	**Value**	.882
		Number of items	20[a]
	Part 2	Value	.839
		Number of items	20[b]
	Total term		40
Correlation between tables			.654
Spearman-Brown coefficient	Equal length		.791
	Unequal length		.791
Guttman Split-Half coefficient			.785

Supplementary Table S12 *Criteria for classification and classification of papers*

**Type of paper**		Assignment
**SCI**	JCR Q1	60
	JCR Q2	21
	JCR Q3	11

	JCR Q4	7
SSCI		30
EI	Periodical	8
	Conference	3
CSSCI		4
Core Journal		1

Supplementary Figure S3 *The direct output of AMOS*

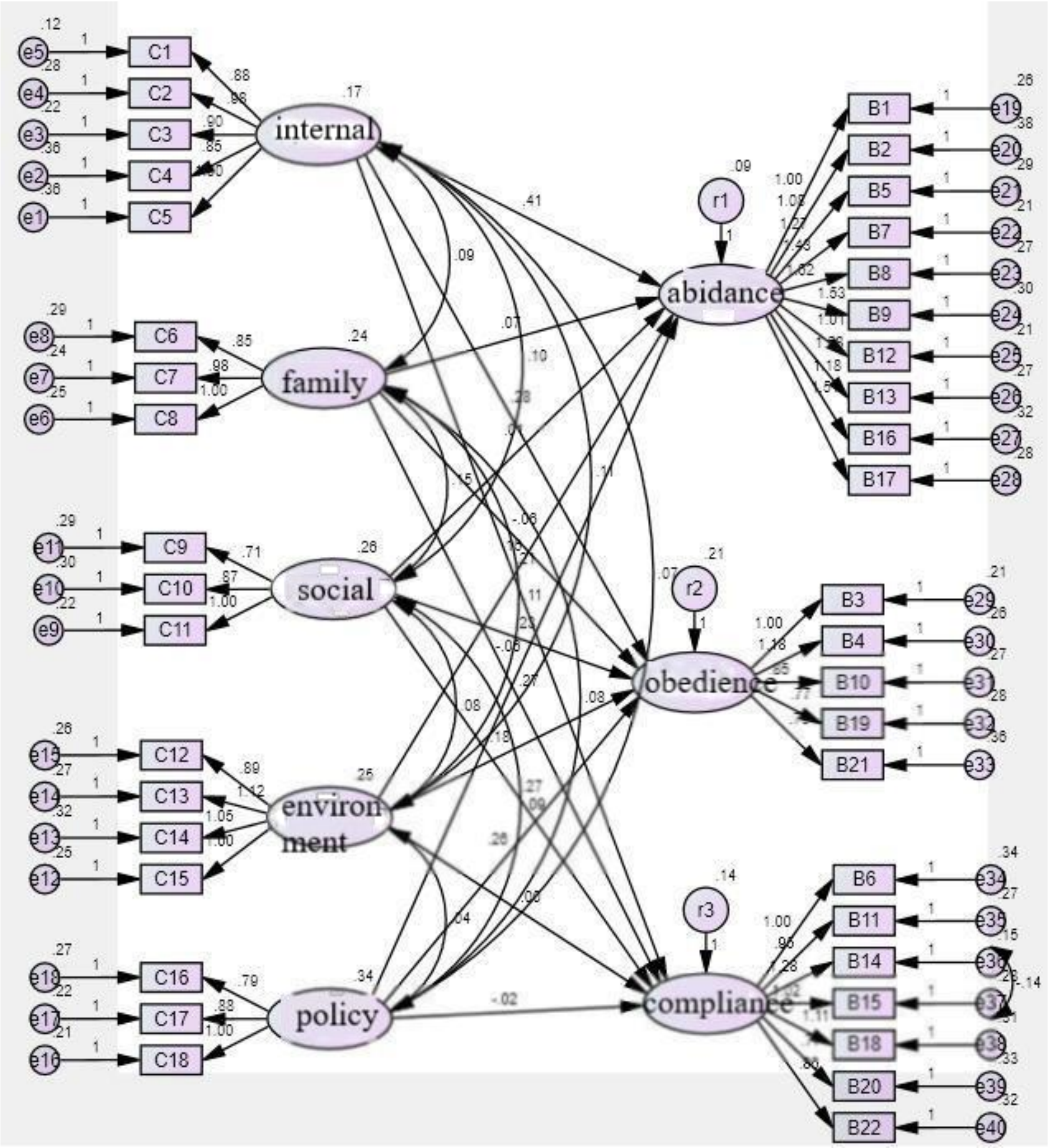

Supplement: Supplementary file 1 [file brainsci-12-01302-s001.zip › brainsci-1886054-supplementary.pdf]
